# Vitamin B6 Status among Vegetarians: Findings from a Population-Based Survey

**DOI:** 10.3390/nu13051627

**Published:** 2021-05-12

**Authors:** Paula Schorgg, Till Bärnighausen, Sabine Rohrmann, Aedin Cassidy, Nena Karavasiloglou, Tilman Kühn

**Affiliations:** 1 Medical Faculty, University of Heidelberg, 69120 Heidelberg, Germany; Schorgg@stud.uni-heidelberg.de; 2 Heidelberg Institute of Global Health (HIGH), University of Heidelberg, 69120 Heidelberg, Germany; till.baernighausen@uni-heidelberg.de; 3 Division of Chronic Disease Epidemiology, Epidemiology, Biostatistics and Prevention Institute (EBPI), University of Zurich, 8001 Zurich, Switzerland; sabine.rohrmann@uzh.ch (S.R.); nena.karavasiloglou@uzh.ch (N.K.); 4 Institute for Global Food Security, Queen’s University Belfast, Belfast BT9 5DL, UK; a.cassidy@qub.as.uk

**Keywords:** vitamin B6, pyridoxal-5′-phosphate, 4-pyridoxic acid, vegetarian diet, population-based

## Abstract

Vitamin B6 from plant foods may have lower bioavailability than vitamin B6 from animal foods, but studies on objectively measured vitamin B6 status among vegetarians compared to non-vegetarians are lacking. Thus, the vitamin B6 status among vegetarians, but also pescatarians, and flexitarians, compared to meat-eaters was assessed in the population-based NHANES study (cycles 2007–2008 and 2009–2010). Data on serum pyridoxal-5′-phosphate (PLP) and 4-pyridoxic acid (4-PA) measured by high-performance liquid chromatography (HPLC) as well as dietary intakes from 24-h recalls were available for 8968 adults aged 20–80 years. Geometric mean (±standard error) PLP concentrations were 58.2 ± 6.0, 52.1 ± 3.7, 49.2 ± 4.6 and 51.0 ± 1.1 nmol/L among vegetarians, pescatarians, flexitarians, and meat-eaters. The 4-PA concentrations were 32.7 ± 4.0, 29.0 ± 2.5, 34.8 ± 5.6 and 33.0 ± 0.7, respectively. There were no statistically significant differences in PLP, 4-PA, and their ratio across the groups in multivariable linear regression models. Overall, the use of vitamin B6 supplements was the strongest predictor of the vitamin B6 status, followed by the dietary vitamin B6 intake. Interestingly, several other covariates were significantly associated with vitamin B6 biomarker levels, particularly serum albumin, creatinine and alkaline phosphatase, and should be considered when assessing the vitamin B6 status. In summary, our findings suggest that a vegetarian diet does not pose a risk for vitamin B6 deficiency.

## 1. Introduction

Plant-based diets avoiding meat and fish, such as a vegetarian diet, have gained popularity over recent years. The plant-based diet trend is promoted by evidence on health risks associated with the consumption of red meat [1,2], but also by environmental and ethical considerations [3].

Compared to meat-eaters, vegetarians are usually younger, more likely to be female, have a higher educational attainment, and show a more health-conscious lifestyle pattern characterized by higher physical activity, non-smoking, and lower body mass index [4,5,6]. While vegetarians may have lower risks for ischemic heart disease, diabetes, total cancer, and eye cataract [7,8], the supply of certain nutrients may be critical with unbalanced vegetarian diets [9].

With respect to the vitamin B6 status, there is evidence to suggest reduced bioavailability [10] as well as digestibility [11] of vitamin B6 from plant foods compared to animal foods. However, previous studies on the vitamin B6 status among vegetarians are inconsistent, reporting lower [12,13] or similar [14,15] serum concentrations of pyridoxal-5′-phosphate (PLP) among vegetarians compared to non-vegetarians. One reason for the heterogeneous data on the vitamin B6 status among vegetarians may be that biomarkers of the vitamin B6 status, especially PLP, but also 4-pyridoxic acid (4-PA), are influenced by a range of potential confounders, such as obesity, smoking or inflammation [16,17], for which significant differences between vegetarians and non-vegetarians have been observed [6,18]. A comprehensive statistical adjustment for these factors has been missing in previous studies on the vitamin B6 status among vegetarians.

In the present study, the vitamin B6 status of vegetarians, pescatarians (omitting meat, but not fish), flexitarians (following a primarily but not strictly vegetarian diet, occasionally eating meat or fish [19]), and meat-eaters was compared in the population-based NHANES. Making use of the unique availability of comprehensive data on lifestyle factors and biomarkers in NHANES, the specific aim was to account for known determinants of the vitamin B6 status beyond diet (e.g., obesity, smoking, alcohol intake, but also kidney function and inflammation) [17] in multivariable analyses on the vitamin B6 status according to the type of diet.

## 2. Materials and Methods

### 2.1. Study Population

NHANES is a study program conducted by the Centers for Disease Control and Prevention’s (CDC) National Center for Health Statistics (NCHS). The objective of the study is to assess the health and nutritional status of the population of the United States (U.S.). The survey population is recruited through a multistage, national area probability design and represents the non-institutionalized civilian U.S. population. Annually, about 5000 participants from 15 counties across the country are included. The survey data are released in cycles of two years, and the data collection comprises interviews and physical examinations; the former targeting lifestyle factors, socio-economic and demographic characteristics as well as dietary habits, the latter focusing on medical conditions, physiological measures and laboratory analyses [20]. Blood samples are collected by a phlebotomist, who also determines the fasting status of the respective participant [21].

The present study incorporated the public use files of the two NHANES cycles 2007–2008 [22] and 2009–2010 [23]. Data files were downloaded from the NHANES website (https://www.cdc.gov/nchs/nhanes/index.htm, last accessed 3 January 2021) and merged into one dataset. All participants provided a written informed consent and the study was approved by the NCHS Research Ethics Review Board (ERB) [24].

### 2.2. Nutritional Assessment

Participants’ self-reports on the type of diet they followed (vegetarian vs. non-vegetarian) were used as the basis for classifying dietary preferences. If the answer to the questionnaire item “Do you consider yourself to be a vegetarian?” was “No” the participant was classified as “meat-eater”, and if the answer was “Yes” as a “vegetarian”. Data from two 24-h recalls per participant [25] and from a specific questionnaire on fish and shellfish consumption in a period of 30 days prior to the examinations were used to assess whether self-perceived vegetarians consumed animal products. Self-perceived vegetarians, who reported to consume seafood, but not meat, were classified as “pescatarians”, and self-perceived vegetarians, who did not report to consume meat and fish were classified as “vegetarians”. As only two self-perceived vegetarians reported not to consume meat, fish, eggs and dairy products at all, no separate category for vegans was created. Self-perceived vegetarians, who reported to consume meat, were classified as “flexitarians”.

The vitamin B6 status was examined on the basis of the serum concentrations of PLP (nmol/L) and 4-PA (nmol/L), as well as their ratio 4-PA/PLP. Both biomarkers were measured by high-performance liquid chromatographic (HPLC) analysis for which further methodological details can be found in the laboratory procedure manual provided by NHANES [26]. PLP, the active co-enzyme form of vitamin B6, is converted into pyridoxal, the vitamins transport form, and then catabolized to 4-PA which is excreted in the urine [17]. Thus, the ratio between the molecules in the blood serum can be used to indicate a reinforced vitamin B6 catabolism or shifted tissue distribution [27].

### 2.3. Covariates

Covariates for multivariable statistical models on the vitamin B6 status across vegetarians, pescatarians, flexitarians, and meat-eaters were selected by literature review [16,17]. Data on the following self-reported covariates were used: demographic characteristics (sex, age, ethnicity, educational attainment, marital status, income), disease prevalence (cardiovascular diseases, diabetes, cancer, liver conditions), and reproductive health items among female participants (pregnancy status, oral contraceptive use (OC), menopausal status). Information on the use of vitamin B6 containing supplements and prescription drugs, which may affect the vitamin B6 status, was also self-reported for the period 30 days prior to the examinations. Based on a literature search, drugs containing the following agents were selected as covariates: non-steroidal anti-inflammatory drugs (NSAIDs), selective COX2 inhibitors, OC, hydralazine, penicillamine, isoniazid, phenelzine, levodopa, and theophylline [17].

Smoking was classified based on the self-reported smoking habits as “non-smoker” (smoked < 100 cigarettes in life), “current smoker” (currently smoking every or some days) and “former smoker” (smoked > 100 cigarettes in life but currently not smoking) in similar manner as previously described by Karavasiloglou et al. [4]. Self-reported physical activity was categorized as previously suggested by Vásquez et al. [28] into levels of “moderate/vigorous” and “none”. According to their self-reported alcohol consumption, participants were classified as “non-drinker”, “moderate drinker”, “binge drinker” and “heavy drinker” in a sex-specific manner, as proposed by Agrawal et al. [29].

Body mass index (BMI; kg/m^2^) was obtained by trained study personnel. Finally, the following serum parameters were included as covariates: C-reactive protein (inflammation), cotinine (active and passive smoking), glycosylated hemoglobin (sugar metabolism), albumin, creatinine (kidney function), and alkaline phosphatase (kidney and liver function).

### 2.4. Statistical Analyses

For descriptive statistical analyses, survey-weighted frequencies (categorical variables) and survey-weighted geometric mean values and standard error (SE) (continuous variables) were used.

Survey-weighted linear regression models were used to evaluate the associations between the vitamin B6 status (PLP, 4-PA, and the ratio of both biomarkers) and the type of diet (vegetarian vs. pescatarian vs. flexitarian and vs. meat-containing). Model 1 was adjusted for age, sex and fasting duration prior to the blood draw. Model 2 was additionally adjusted for educational attainment, ethnicity, BMI, smoking status, alcohol consumption, physical activity, prevalent diabetes mellitus, prevalent liver conditions, history of cancer, history of cardiovascular disease, vitamin B6 supplement use, dietary vitamin B6 intake, current OC use, menopausal status, prescription drug usage (theophylline, l-dopamine, non-steroidal anti-inflammatory drugs, COX2-inhibitors, isoniazid or hydralazine), and serum parameters (C-reactive protein, cotinine, glycosylated hemoglobin, albumin, creatinine, and alkaline phosphatase).

Associations were considered as statistically significant at two-sided *p*-values < 0.05. Semi-partial-R^2^ values were calculated to assess the strengths of the associations between covariates and vitamin B6 biomarkers. For all statistical analyses SAS 9.4 (Cary, NC, USA) was used.

## 3. Results

### 3.1. Characteristics of the Study Population

**Figure A1:**
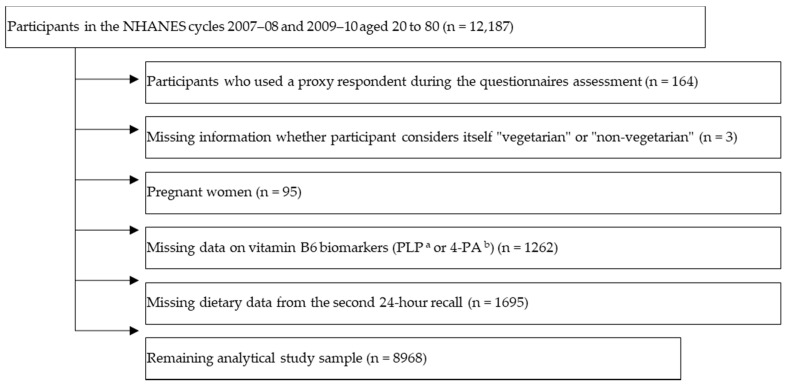
Characteristics and exclusion of study population. ^a^ Pyridoxal-5′-Phosphate; ^b^ 4-Pyridoxic Acid.

Overall, 12,187 adults aged 20 to 80 years participated in the NHANES cycles 2007–2008 and 2009–2010. We excluded participants, who had their questionnaires answered by proxy respondents (n = 164), who did not provide information on whether they were self-perceived vegetarian or non-vegetarian (n = 3), or who were pregnant (n = 95). Moreover, 1262 participants were excluded due to missing vitamin B6 status parameters (PLP or 4-PA), and 1695 were excluded due to missing dietary data from the second 24-h recall assessment. Thus, the analytical sample consisted of 8968 participants (Appendix A Figure A1).

Characteristics of the study population are shown in Table 1. The vegetarian, pescatarian, and flexitarian groups consisted of a larger proportion of women (62.5%, 65.7%, and 61.6%, respectively) than the meat-eaters (51.6%). The mean age of vegetarians (32.1 (SE: 1.2)) and pescatarians (38.8 (1.8)) was approximately 10 years lower than the mean age of the flexitarians (47.4 (2.5)) and the meat-eaters (44.7 (0.4)). Educational attainment was higher among vegetarians and pescatarians compared to flexitarians and meat-eaters, and vegetarians generally showed a more favorable pattern of lifestyle factors, with higher prevalence of physical activity and non-smoking. As expected, vegetarians consumed higher amounts of fruits, vegetables and fiber compared to meat-eaters (Appendix A Table A1). They also showed lower concentrations of cotinine and alkaline phosphatase in serum compared to meat-eaters (Appendix A Table A2).

### 3.2. Vitamin B6 Levels across the Groups

**Figure 1:**
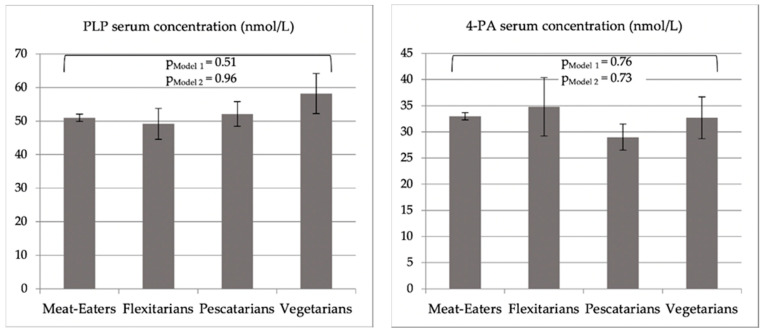

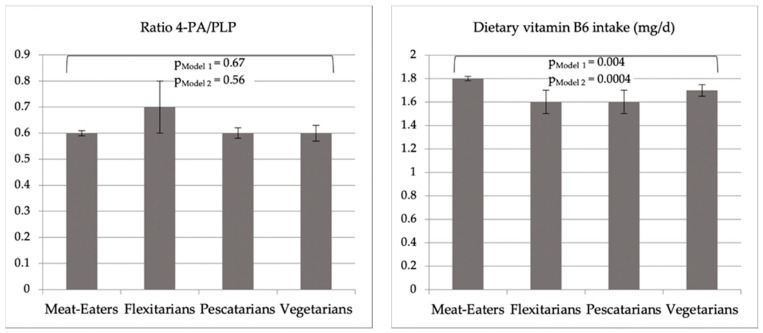
Geometric mean values of PLP (pyridoxal-5’-phosphate) and 4-PA (4-pyridoxic acid) serum concentrations (nmol/L), their ratio, and total vitamin B6 intake (mg/d) of study participants following a meat-eating, flexitarian, pescatarian or vegetarian diet. P_Model 1_/P_Model 2_: *p*–values shown for overall differences across all groups from linear regression Models 1 and 2; the only pairwise differences between groups that were statistically significant were those in vitamin B6 intake between vegetarians and non-vegetarians at p-values of 0.0027 and <0.0001 in Model 1 and Model 2, respectively. Model 1 *: Adjusted for age, sex and fasting duration before blood draw; Model 2 *: further adjusted for educational attainment, ethnicity, body mass index, smoking status, alcohol consumption, physical activity, diabetes mellitus prevalence, cancer prevalence, cardiovascular disease prevalence, vitamin B6 supplement use, dietary vitamin B6 intake, liver condition prevalence, current oral contraceptive use, menopausal status, prescription drug usage (theophylline, l-dopamine, non-steroidal anti-inflammatory drugs, COX2-inhibitors, isoniazid and hydralazine), and serum parameters (C-reactive protein (CRP), cotinine, glycosylated hemoglobin, albumin, creatinine, alkaline phosphatase). Due to missing biomarker values among 83 participants overall (n missing for CRP = 9, cotinine = 23, glycosylated hemoglobin = 15, albumin = 62, creatinine = 63, alkaline phosphatase = 63) Model 2 included data of 8885 participants. * Models on dietary vitamin B6 not adjusted for fasting duration; Table A3 (Appendix A) further shows adjusted geometric mean vitamin B6 vitamer serum concentrations obtained by the least squares means method.

Vegetarians reported the most frequent use of dietary supplements containing vitamin B6 (44.6% vs. 31.8% among meat-eaters) (Table 1). Mean dietary vitamin B6 intakes (SEs) were 1.7 (0.05), 1.6 (0.1), 1.6 (0.1) and 1.8 (0.02) mg/d among vegetarians, pescatarians, flexitarians and meat-eaters, respectively. These differences were not statistically significant in a “crude” unadjusted regression model. However, linear regression analyses adjusted for age and sex showed a statistically significant difference in average dietary vitamin B6 intakes across all groups (Model 1, Figure 1, *p* = 0.004), driven by significantly lower levels of vegetarians compared to meat-eaters (at *p* = 0.0027), while no pairwise differences between the other groups were observed. These overall (Model 2, Figure 1 and Appendix A Table A3, *p* = 0.004) and pairwise differences between vegetarians and meat-eaters (*p* < 0.0001) remained statistically significant upon multivariable adjustment.

Mean vitamin B6 vitamer concentrations were only slightly different across the groups (Table 1). The mean PLP concentrations were highest in the vegetarian group (58.2 (6.0) nmol/L) and lowest in the flexitarian group (49.2 (4.6) nmol/L). All groups had mean PLP concentrations above the recommended value of 20 nmol/L [30]. PLP values <20 nmol/L indicative of vitamin B6 deficiency were observed among 16.1%, 5.7%, 11.6% and 14.7% of the vegetarians, pescatarians, flexitarians and meat-eaters, respectively (Appendix A Table A4). The 4-PA mean concentration was highest in the flexitarian group (34.8 (5.6) nmol/L) and lowest in the pescatarian group (29.0 (2.5) nmol/L). The ratio of 4-PA/PLP was highest in the flexitarian group (0.7 (0.1)) and similar amongst vegetarians (0.6 (0.03)), pescatarians (0.6 (0.02)) and meat-eaters (0.6 (0.01)).

There were no statistically significant differences in PLP, 4-PA, or 4-PA/PLP across the four groups in linear regression analyses adjusted for age, sex, and fasting duration (Model 1) or following further adjustment for a wider range of confounders including vitamin B6 supplement use (Model 2, see Figure 1 and Table A3). Sensitivity analyses among the study participants, who did not use vitamin B6 supplements, did not show any differences in PLP, 4-PA, or 4-PA/PLP across the groups either.

### 3.3. Predictors of Vitamin B6 Status

To estimate the impact of the covariates on vitamin B6 vitamer serum concentrations, semi-partial-R^2^ values were calculated (Table 2).

PLP serum concentration was strongly associated with the use of vitamin B6 containing dietary supplements (18.1% of the variance in PLP), dietary vitamin B6 intake (5.4%), alkaline phosphatase levels (4.0%), and albumin levels (3.4%). 4-PA was also most strongly associated with vitamin B6 supplement use (18.5%), followed by creatinine levels (5.0%), dietary vitamin B6 intake (4.2%), and age (2.1%). The ratio of 4-PA/PLP was most strongly associated with creatinine levels (8.2%), sex (1.6%), and albumin levels (1.0%).

## 4. Discussion

In the present study, no differences in biomarkers of the vitamin B6 status (serum PLP, 4-PA, and their ratio, i.e., 4-PA/PLP) were observed between vegetarians, pescatarians, flexitarians, and meat-eaters in the NHANES, despite the slightly lower dietary vitamin B6 intake among vegetarians. The majority of study participants across all groups reached the recommended dietary allowance (RDA) values for vitamin B6 intake of 1.3 mg/d and 1.5 mg/d for women aged 19–50 years and >51 years, respectively, and 1.3 mg/d and 1.7 mg/d for men aged 19–50 years and >51 years [30]. These data are important as they provide evidence that a vegetarian diet does not pose a risk for vitamin B6 deficiency. In addition to supplemental vitamin B6 intake and dietary vitamin B6 intake, several lifestyle factors (including age, sex, ethnicity) and biochemical parameters (particularly creatinine, albumin, alkaline phosphatase) were significantly associated with the vitamin B6 status. While many of these determinants of the vitamin B6 status were not equally distributed among the four groups, with a higher prevalence of vitamin B6 supplement use among vegetarians, for example, a multivariable statistical adjustment for a broad range of confounders did not affect our results on vitamin B6 levels across the diet groups.

The lack of a significant difference in PLP blood concentrations between vegetarians compared to meat-eaters is in line with observations from two studies among Buddhist nuns from Taiwan and adult Austrian volunteers [14,15]. Another study among young adults from Taiwan found PLP values indicating vitamin B6 sufficiency among both vegetarians and non-vegetarians, although the vegetarian group showed significantly lower mean PLP concentrations (58.5 vs. 85.9 nmol/L) [12]. Lower PLP concentrations among adult vegetarians (with 58% showing a deficiency) compared to omnivores were reported from Switzerland [13]. However, vegan participants of this study showed higher average PLP concentrations (27 nmol/L) than both omnivores (22 nmol/L) and vegetarians (16 nmol/L). The observation of much higher PLP values across all groups in the present study compared to the Swiss study may be explained by the wide-spread use of dietary vitamin B6 supplements in the NHANES population (31.90%). Overall, despite potentially lower bioavailability of vitamin B6 from plant sources [10,11], ours together with previous epidemiological studies do not point to a generally lower vitamin B6 status among vegetarians compared to non-vegetarians.

Interestingly, and for the first time in a large-scale epidemiological study, to our knowledge, we showed that a wide range of lifestyle factors, prevalent medical conditions, medications, and biochemical indicators are associated with both PLP and 4-PA concentrations as well as their ratio. As expected, the vitamin B6 intake, particularly via supplements but also via food, was the strongest determinant of the vitamin B6 status. Furthermore, as reported from previous smaller studies, albumin and alkaline phosphatase concentrations are associated with PLP concentrations [17]. Regarding 4-PA, creatinine and age were important determinants, which is consistent with elevated serum 4-PA among patients suffering from renal insufficiency [31]. Overall, our population-based study underlines that these factors should be taken into account when measuring PLP and 4-PA concentrations in studies on the vitamin B6 status and disease risks [17].

The NHANES dataset we used provided unique advantages, in particular regarding the extensive data collection. Thus, we had the opportunity to incorporate a comprehensive set of determinants of the vitamin B6 status, which showed differential distributions across vegetarians and non-vegetarians, into our multivariable analyses. Limitations of our analyses include that the Par index (4-PA/ [pyridoxal + PLP]), a sensitive alternative indicator to evaluate the vitamin B6 metabolism [27], was not available, as pyridoxal was not measured in NHANES. Moreover, as in most epidemiological studies, our classification of the dietary patterns was based on self-perception and self-reported dietary information at one point in time. However, the sociodemographic characteristics and the more favorable pattern of lifestyle factors of vegetarians in our study is in line with reports from previous studies [5,6], and the combination of assessment tools to derive the groups may have prevented misclassification. We did not have the opportunity to assess vitamin B6 status among vegans, although two smaller studies from Austria and Switzerland do not point to a lower vitamin B6 status among vegans compared to omnivores [13,15]. The high proportion of vitamin B6 supplement users in NHANES may not be comparable to proportions in other populations. Nevertheless, statistical adjustment for supplement use did not change our main finding, i.e., the lack of a difference in biomarkers of the vitamin B6 status across diet groups.

To conclude, in the present analysis no evidence was found that the type of diet (vegetarian, pescatarian, flexitarian, and meat-eating) consumed affects vitamin B6 serum concentrations. Therefore, meat and fish, which are good sources of vitamin B6 [32], may not be needed to achieve a sufficient vitamin B6 status, and vitamin B6 may not be a critical nutrient among vegetarians.

## Figures and Tables

**Table 1:** Survey-weighted general characteristics of NHANES 2007–2010 study participants following a meat-eating, flexitarian, pescatarian or vegetarian diet (n = 8968).

	Unweighted Participants Counts (Survey-Weighted Frequencies)			
	Meat-Eaters 8765 (97.7)	Flexitarians 112 (1.3)	Pescatarians 35 (0.4)	Vegetarians 56 (0.6)
Age, mean ^a^ (SE)	44.7 (0.4)	47.4 (2.5)	38.8 (1.8)	37.1 (1.2)
Sex, n (%)				
Female	4519 (51.6)	69 (61.6)	23 (65.7)	35 (62.5)
Male	4246 (48.4)	43 (38.4)	12 (34.3)	21 (37.5)
Educational attainment, n (%)				
College or higher	4229 (48.2)	53 (47.3)	23 (65.7)	49 (87.5)
High school or lower	4536 (51.8)	59 (52.7)	12 (34.3)	7 (12.5)
Ethnicity, n (%)				
Mexican American	1494 (17.0)	21 (18.8)	2 (5.7)	5 (8.9)
Non-Hispanic Black	1610 (18.4)	17 (15.2)	5 (14.3)	2 (3.6)
Non-Hispanic White	4427 (50.5)	40 (35.7)	21 (60.0)	29 (51.8)
Other	1234 (14.1)	34 (30.4)	7 (20.0)	20 (35.7)
Body mass index (kg/m^2^), mean (SE)	28.1 (0.1)	26.1 (0.4)	25.4 (0.7)	24.9 (0.5)
Smoking status, n (%)				
Current smoker	1884 (21.5)	9 (8.0)	3 (8.6)	6 (10.7)
Former smoker	2254 (25.7)	29 (25.9)	11 (31.4)	11 (19.6)
Non-smoker	4627 (52.8)	74 (66.1)	21 (60.0)	39 (69.6)
Alcohol consumption, n (%)				
Binge or Heavy drinker	2837 (32.4)	24 (21.4)	14 (40.0)	15 (26.8)
Moderate drinker	2686 (30.6)	24 (21.4)	9 (25.7)	18 (32.1)
Non drinker	1654 (18.9)	26 (23.2)	5 (14.3)	6 (10.7)
Unknown/missing	1588 (18.1)	38 (33.9)	7 (20.0)	17 (30.4)
Physical activity, n (%)				
Moderate or vigorous	3969 (45.3)	43 (38.4)	25 (71.4)	35 (62.5)
None	4796 (54.7)	69 (61.6)	10 (28.6)	21 (37.5)
Vitamin B6 vitamers, mean (SE)				
PLP ^b^ (nmol/L)	51.0 (1.1)	49.2 (4.6)	52.1 (3.7)	58.2 (6.0)
4-PA ^c^ (nmol/L)	33.0 (0.7)	34.8 (5.6)	29.0 (2.5)	32.7 (4.0)
Ratio, 4-PA/PLP	0.6 (0.01)	0.7 (0.1)	0.6 (0.02)	0.6 (0.03)
Dietary vitamin B6 Supplement Use ^d^, n (%)				
No	5975 (68.2)	79 (70.5)	22 (62.9)	31 (55.4)
Yes	2790 (31.8)	33 (29.5)	13 (37.1)	25 (44.6)
Dietary vitamin B6 Intake (mg/d), mean (SE)	1.8 (0.02)	1.6 (0.1)	1.6 (0.1)	1.7 (0.05)
Total ^e^ vitamin B6 Intake (mg/d), mean (SE)	2.8 (0.1)	2.5 (0.3)	3.3 (0.9)	2.9 (0.3)

^a^ Mean values are geometric mean values; ^b^ PLP = pyridoxal-5′-phosphate; ^c^ 4-PA = 4-pyridoxic acid; ^d^ includes vitamin B6 from multivitamin and micronutrient preparations; ^e^ Total vitamin B6 intake includes dietary and supplementary vitamin B6.

**Table 2:** Predictors of vitamin B6 vitamer serum concentrations (n = 8885) ^a^.

	Pyridoxal-5′-Phosphate		4-Pyridoxic Acid		Ratio, 4-PA/PLP	
	R^2^	*p*	R^2^	*p*	R^2^	*p*
Diet Type	<0.1	0.96	<0.1	0.73	<0.1	0.56
Age	<0.1	0.16	2.1	<0.0001	0.1	0.003
Sex	0.1	0.44	0.8	0.06	1.6	0.003
Fasting duration	0.3	0.0013	1.2	<0.0001	0.3	<0.0001
Vitamin B6 supplement use	18.1	<0.0001	18.5	<0.0001	0.7	<0.0001
Dietary vitamin B6 intake	5.4	<0.0001	4.2	<0.0001	<0.1	0.91
Body mass index	0.7	<0.0001	0.3	0.0008	0.1	0.04
Educational attainment	<0.1	0.055	<0.1	0.30	<0.1	0.38
Ethnicity	0.2	<0.0001	1.5	<0.0001	0.2	0.002
Smoking status	0.1	0.14	<0.1	0.38	0.1	0.07
Alcohol consumption	0.1	0.06	0.1	0.31	<0.1	0.29
Physical activity	0.2	0.01	<0.1	0.24	0.1	0.02
Prevalent diabetes mellitus	0.2	0.001	0.1	0.32	0.3	0.02
History of cancer	0.1	0.11	<0.1	0.92	<0.1	0.33
History cardiovascular diseases	<0.1	0.23	<0.1	0.10	0.1	0.02
Prevalent liver disease	0.1	0.08	<0.1	0.34	<0.1	0.02
Oral contraceptive use	0.1	0.03	0.1	0.35	<0.1	0.10
Menopausal status	0.8	<0.0001	0.1	0.04	0.1	0.24
Prescription drug use						
Theophylline	0.1	0.0015	<0.1	0.15	0.4	0.02
L-Dopamin	<0.1	0.30	<0.1	0.08	<0.1	0.18
NSAID	<0.1	0.25	<0.1	0.64	<0.1	0.43
COX2 Inhibitors	0.1	0.006	0.1	0.01	<0.1	0.31
Isoniazid	0.1	0.0001	0.1	<0.0001	<0.1	0.93
Hydralazine	<0.1	0.27	<0.1	0.71	<0.1	0.32
Serum parameters						
C-reactive protein	0.2	0.0096	<0.1	0.22	0.1	<0.0001
Cotinine	0.7	<0.0001	0.5	<0.0001	<0.1	0.25
Glycohemoglobin	0.1	0.02	0.1	0.007	0.2	0.002
Albumin	3.4	<0.0001	0.1	0.10	1.0	<0.0001
Creatinine	0.3	0.0002	5.0	<0.0001	8.2	<0.0001
Alkaline phosphatase	4.0	<0.0001	0.1	0.04	0.8	<0.0001
Model R^2^	40.98		39.33		16.50	

^a^ Semi-partial R^2^ values from a multivariable linear regression model mutually adjusted for all variables in the table. Due to missing biomarker values among 83 participants (n missing for CRP = 9, cotinine = 23, glycosylated hemoglobin = 15, albumin = 62, creatinine = 63, alkaline phosphatase = 63) Model 2 included data of n = 8885 participants.

## Data Availability

Data of the National Health and Nutrition Examination Survey including those used for the present study can be downloaded on the homepage of the Centers of Disease Control and Prevention (https://wwwn.cdc.gov/nchs/nhanes/Default.aspx accessed on 1 December 2020).

## Appendix A

**Table A1:** Survey-weighted food group consumption of NHANES 2007–2010 study participants following a meat-eating, flexitarian, pescatarian or vegetarian diet.

	Unweighted Participants Counts (Survey-Weighted Frequencies)			
	Meat-Eaters 8765 (97.7)	Flexitarians 112 (1.3)	Pescatarians 35(0.4)	Vegetarians 56 (0.6)
Dietary intake, median (IQR)				
Meat (oz. eq.)	1.2 (2.5)	0.2 (1.2)	0.0 (0.0)	0.0 (0.0)
Seafood (oz. eq.)	0.0 (0.3)	0.0 (0.2)	0.4 (0.9)	0.0 (0.0)
Poultry (oz. eq.)	1.0 (2.4)	0.7 (1.7)	0.0 (0.0)	0.0 (0.0)
Fruit (cup eq.)	0.7 (1.4)	0.9 (1.2)	1.0 (1.3)	1.1 (1.8)
Vegetables and legumes ^a^ (cup eq.)	0.5 (0.5)	0.6 (0.6)	0.7 (0.6)	0.7 (0.9)
Dairy (cup eq.)	1.4 (1.4)	1.0 (1.2)	1.2 (1.7)	1.3 (1.7)
Eggs (oz. eq.)	0.2 (0.8)	0.2 (0.7)	0.1 (0.7)	0.1 (0.2)
Dietary fiber (g/d)	15.2 (10.1)	15.8 (14.0)	22.8 (14.7)	22.4 (18.2)

^a^ Legumes include starchy and protein computed legume.

**Table A2:** Survey-weighted serum parameters of NHANES 2007–2010 study participants following a meat-eating, flexitarian, pescatarian or vegetarian diet (n = 8885 ^a^).

	Unweighted Participants Counts (Survey-Weighted Frequencies)			
	Meat-Eaters	Flexitarians	Pescatarians	Vegetarians
Serum parameters, mean ^b^ (SE)				
C-reactive protein(mg/dL)	0.2 (0.004)	0.1 (0.01)	0.1 (0.02)	0.1 (0.02)
Cotinine (ng/mL)	0.3 (0.03)	0.1 (0.04)	0.1 (0.02)	0.2 (0.1)
Glycosylated hemoglobin (%)	5.6 (0.01)	5.7 (0.07)	5.3 (0.04)	5.4 (0.045)
Albumin (g/dL)	4.3 (0.007)	4.3 (0.03)	4.2 (0.1)	4.3 (0.046)
Creatinine (mg/dL)	0.9 (0.005)	0.8 (0.02)	0.7 (0.02)	0.8 (0.02)
Alkaline phosphotase (U/L)	64.1 (0.3)	64.1 (2.9)	62.3 (4.6)	60.3 (2.4)

^a^ Due to missing biomarker values among 83 participants overall (n missing for CRP = 9, cotinine = 23, glycosylated hemoglobin = 15, albumin = 62, creatinine = 63, alkaline phosphatase = 63) Model 2 included data of 8885 participants. ^b^ Mean values are geometric mean values.

**Table A3:** Adjusted geometric mean vitamin B6 vitamer serum concentrations of study participants following a meat-eating, flexitarian, pescatarian or vegetarian diet ^a^.

	Meat-Eaters	Flexitarians	Pescatarians	Vegetarians	*p* ^b^
	Model 1				
PLP ^c^, mean ± SE	51.1 ± 1.0	50.2 ± 1.1	54.7 ± 1.1	59.6 ± 1.2	0.51
4-PA ^d^, mean ± SE	34.7 ± 1.0	34.4 ± 1.2	35.0 ± 1.2	41.2 ± 1.2	0.76
Ratio, 4-PA/PLP, mean ± SE	2.6 ± 1.0	3.4 ± 1.3	2.4 ± 1.1	2.6 ± 1.0	0.67
	Model 2				
PLP, mean ± SE	70.1 ± 1.2	69.2 ± 1.2	64.5 ± 1.3	69.4 ± 1.2	0.96
4-PA, mean ± SE	76.4 ± 1.4	82.5 ± 1.3	67.1 ± 1.4	81.0 ± 1.4	0.73
Ratio, 4-PA/PLP, mean ± SE	5.9 ± 1.8	8.0 ± 1.9	6.1 ± 1.8	6.4 ± 1.8	0.56

^a^ Mean values obtained by the least squares means method from multivariable adjusted linear regression models; ^b^ *p*-value for overall differences across groups from linear regression models; none of the pairwise between-group comparisons showed differences at *p* < 0; ^c^ PLP = pyridoxal-5′-phosphate; ^d^ 4-PA = 4-pyridoxic acid. Model 1: Adjusted for age, sex and fasting duration before blood draw. Model 2: Adjusted for age, sex, fasting duration before blood draw, educational attainment, ethnicity, body mass index, smoking status, alcohol consumption, physical activity, diabetes mellitus prevalence, cancer prevalence, cardiovascular disease prevalence, vitamin B6 supplement use, dietary vitamin B6 intake, liver condition prevalence, current oral contraceptive use, menopausal status, prescription drug usage (theophylline, l-dopamine, non-steroidal anti-inflammatory drugs, COX2-inhibitors, isoniazid and hydralazine), and serum parameters (C-reactive protein (CRP), cotinine, glycosylated hemoglobin, albumin, creatinine, alkaline phosphatase). Due to missing biomarker values among 83 participants overall (n missing for CRP = 9, cotinine = 23, glycosylated hemoglobin = 15, albumin = 62, creatinine = 63, alkaline phosphatase = 63) Model 2 included data of 8885 participants.

**Table A4:** Survey-weighted categories of vitamin B6 supply across NHANES 2007–2010 study participants following a vegetarian, pescatarian, flexitarian and meat-eating diet based on PLP ^a^ serum concentrations (n = 8968).

	Unweighted Participants Counts (Survey-Weighted Frequencies)			
	Meat-Eaters 8765 (97.7)	Flexitarians 112 (1.3)	Pescatarians 35 (0.4)	Vegetarians 56 (0.6)
Categories, n (%)				
Adequate (PLP > 30 nmol/L)	5987 (68.3)	83 (74.1%)	28 (80.0%)	42 (75.0%)
Moderately deficient (PLP 20–30 nmol/L)	1487 (17.0)	16 (14.3%)	5 (14.3%)	5 (8.9%)
Deficient (PLP < 20 nmol/L)	1291 (14.7)	13 (11.6%)	2 (5.7%)	9 (16.1%)

^a^ PLP = pyridoxal-5′-phosphate.
